# A Compendium of Insanity

**Published:** 1898-05

**Authors:** 


A COMPENDIUM OF INSANITY. By JOHN B. CHAPIN, M. D., LLD., Physician-in-Chief Pennsylvania Hospital for the Insane; late Physician-Superintendent of the Willard State Hospital, New York, etc. Duodecimo, 234 pages. Illustrated. Philadelphia: W. B. Saunders. 1898.

A compend on insanity by one who knows what to insert, and, what is more important, what to omit, has been needed for some time by the general practitioner, members of the legal profession, and by the public at large, enabling them to recognise insane tendencies and to provide intelligently for any case of insanity in the family that may demand urgent care and treatment. Chapin's compendium is just such a book and it can be cheerfully recommended. It gives the clinical aspects of the various abnormal conditions, the treatment and management of the insane. The chapter on Morbid anatomy was prepared by J. Montgomery Mosher, of Albany, N. Y., and is lucidly stated and embraces all the newer ideas and discoveries. A chapter on medical certificates and feigned insanity adds to the value of the book.

The illustrations showing the various forms of insanity are well chosen and nicely executed. The volume is well printed and neatly bound. W. C. K.

